# Autophagy Activator Drugs: A New Opportunity in Neuroprotection from Misfolded Protein Toxicity

**DOI:** 10.3390/ijms20040901

**Published:** 2019-02-19

**Authors:** Stefano Thellung, Alessandro Corsaro, Mario Nizzari, Federica Barbieri, Tullio Florio

**Affiliations:** 1 Sezione di Farmacologia, Dipartimento di Medicina Interna & Centro di Eccellenza per la Ricerca Biomedica (CEBR), Università di Genova, 16132 Genova, Italy, stefano.thellung@unige.it (S.T.); ale.corsaro@unige.it (A.C.); mario.nizzari@unige.it (M.N.); federica.barbieri@unige.it (F.B.); 2 IRCCS Ospedale Policlinico San Martino, 16132 Genova, Italy

**Keywords:** neurodegenerative diseases, protein misfolding, autophagy, mTOR, rapamycin

## Abstract

The aim of this review is to critically analyze promises and limitations of pharmacological inducers of autophagy against protein misfolding-associated neurodegeneration. Effective therapies against neurodegenerative disorders can be developed by regulating the “self-defense” equipment of neurons, such as autophagy. Through the degradation and recycling of the intracellular content, autophagy promotes neuron survival in conditions of trophic factor deprivation, oxidative stress, mitochondrial and lysosomal damage, or accumulation of misfolded proteins. Autophagy involves the activation of self-digestive pathways, which is different for dynamics (macro, micro and chaperone-mediated autophagy), or degraded material (mitophagy, lysophagy, aggrephagy). All neurodegenerative disorders share common pathogenic mechanisms, including the impairment of autophagic flux, which causes the inability to remove the neurotoxic oligomers of misfolded proteins. Pharmacological activation of autophagy is typically achieved by blocking the kinase activity of mammalian target of rapamycin (mTOR) enzymatic complex 1 (mTORC1), removing its autophagy suppressor activity observed under physiological conditions; acting in this way, rapamycin provided the first proof of principle that pharmacological autophagy enhancement can induce neuroprotection through the facilitation of oligomers’ clearance. The demand for effective disease-modifying strategies against neurodegenerative disorders is currently stimulating the development of a wide number of novel molecules, as well as the re-evaluation of old drugs for their pro-autophagic potential.

## 1. Introduction

Neurons, as all long-lived cells, must face a ceaseless flow of damage, including reduction of trophic factor production and/or activity, oxidative damage of cytoplasmic structures, and accumulation of lipofuscins and misfolded proteins. Neuronal survival is hence made possible by the correct functioning of several homeostatic systems that allow the recycling of intracellular materials and degradation of malfunctioning structures.

Among these strategies, autophagy plays a crucial role in removing aggregated proteins and damaged organelles, thus promoting the clearance of cytoplasm from triggers of neuronal death. Autophagy is a process that has been highly conserved throughout eukaryote evolution, being present in yeast, plants, animals, and humans, but a full understanding of its exact biological role still represents a major challenge. In humans, autophagy is a main crossroad between health and disease, although the characterization of autophagy-related structures, as well as the genes and proteins that control its progression, derives from studies of adaptive strategies for environmental change in yeasts [1,2,3]. On the other hand, most of the proteins driving starvation-induced autophagy in yeasts have mammalian orthologues that hold similar roles throughout the process.

Three forms of autophagy—micro- and macro-autophagy, and chaperone-mediated autophagy (CMA)—have been described in mammals, according to the structures involved and the dynamics of sequestration of cytoplasm portions [4,5]. While CMA and, to a certain extent, microautophagy cooperate with ubiquitin-proteasome system (UPS) in targeting the degradation of specific proteins, macroautophagy is directed towards the removal of bulky protein aggregates and cytoplasmic bodies. Macroautophagy, often simply called autophagy, is initiated with the formation of the phagophore which is a cup-shaped double-membrane isolation structure whose edges expand, engulfing portions of cytoplasm along with the material committed to degradation. Phagophore closure produces a double-membrane vesicle called autophagosome or amphisome, in the case it receives material from extracellular space through the contribution of endosomes. Microtubules drive autophagosomes and amphisomes to fuse with lysosomes, thus generating single-membrane autolysosomes and autophagolysosomes, respectively [6]; thus, autophagy is completed when the cargo is degraded and its constituents recycled into cytosol. Further heterogeneity in autophagy is based on the targeted material, rather than on the nature of the structures involved; macroautophagy can be specifically targeted to large cytoplasmic protein complexes or entire organelles, such as peroxisomes, mitochondria, lysosomes, endoplasmic reticulum, and nuclei [7,8,9]. Although all these varieties of macroautophagy are conceptually and structurally similar, they involve different subsets of receptor and adaptor proteins. Particularly relevant in the pathogenesis of most neurodegenerative disorders of the central nervous system (CNS), aggrephagy and mitophagy are targeted to sequester aggregates of misfolded proteins and damaged mitochondria, respectively. It is widely accepted that they accomplish a major cytoprotective task in neurons, since defective autophagy is frequently observed in association with neuronal loss and cognitive decline, both in physiological conditions, such as in aging, and in neurodegenerative CNS [10,11].

This review aims to examine the most recent findings about neuroprotective potential of drugs that have been proposed to exert a pro-autophagy activity, focusing on their mechanism of action, efficacy and toxicity. In particular, between December 2018 and January 2019, we searched the PubMed database for “autophagy” and Alzheimer’s disease (AD, 735 entries), Parkinson’s disease (PD, 893), Huntington’s disease (HD, 262), prion diseases (191), or amyotrophic lateral sclerosis (ALS, 377); in particular, we focused our analysis on studies addressing the pharmacological modulation of autophagy as potential treatment for neurodegenerative diseases.

## 2. Protein Conformational Disorders (PCDs)

AD, PD, HD, ALS, and transmissible spongiform encephalopathies (TSE or prion diseases) are neurodegenerative disorders that differ greatly in terms of etiology, histopathology and clinical presentation. Nevertheless, a recent theory claims that all these neurodegenerative diseases recognize the conformational misfolding of specific proteins, whose function in the brain is often obscure, as a common mechanism of neurotoxicity [12]. Among these disorders, the differences in the mechanisms by which protein misfolding occurs may account for the heterogeneous etiology of the diseases; however, neuronal loss is likely caused by the generation of soluble protein oligomers along the process of aggregation and amyloidogenesis that follows the misfolding [13]. Another important pathogenic event that unifies PCDs and, to a certain extent, also occurs during aging, is represented by autophagy alterations and impaired proteostasis [14]. Efficient proteasome-mediated protein quality control cooperates with autophagy to induce neuroprotection: the former process prevents the generation of misfolded proteins, while the latter allows neurons to reduce their intracellular burden of aggregating oligomers, eliminating damaged intracellular structures [11,15]. A promising outcome of such unifying pathogenic hypothesis regards the possibility to set up a common neuroprotective strategy against several different forms of CNS amyloidosis through the pharmacological enhancement or restoration of the autophagic flow [16].

## 3. Protein Misfolding Generates Neurotoxic Oligomers

The deposition of amyloid protein as extracellular plaques and fibrils or intracellular bodies, and neurofibrillary tangles are associated with synaptic rarefaction, glial proliferation and neuronal death [17]. This evidence, mainly derived from AD research, underscores the amyloid theory, suggesting that misfolded β-amyloid (Aβ) aggregates represent the AD neurotoxic entity. This hypothesis led to several attempts to develop disease-modifying therapies aimed at removing amyloid aggregates from the brain or the prevention of their maturation. The overall failure of these approaches, together with the observation that increasing the burden of amyloid plaques in the brain can also be associated with healthy aging, led to the amplification and re-modulation of the amyloid theory, rather than a confutation [18]. The most striking hint arises from the observation that brains from AD patients differ from those of non-demented individuals with respect to the number of Aβ peptides in the form of soluble oligomers [19,20]; aggregates of Aβ oligomers have also been described as extracellular cluster at early stages of AD, and precede the detection of amyloid plaques in animal models of the disease [21]. Finally, the emergence of transmissible spongiform encephalopathies (TSEs), or prion diseases, introduced a definitive revolution in the scientific approaches to nearly all proteinopathy-associated neurodegenerative diseases [22]. Prion diseases are progressive neurodegenerative disorders that afflict humans and animals, with sporadic, inherited and acquired modalities; these pathological conditions are associated with an amyloidogenic misfolding of a normal glycoprotein, called cellular prion protein (PrP^C^), into a protease-insensitive isoform named prion protein Scrapie (PrP^Sc^). Although the associated symptomatology may significantly differ on the basis of the specific brain areas that are primarily involved with PrP^Sc^ deposition, there is a spatial and temporal correlation between deposition of PrP^Sc^ and synaptic rarefaction, glial reaction and grey matter vacuolation. Different molecular events favor PrP^C^-PrP^Sc^ misfolding and account for the heterogeneity in prion diseases etiology: the conversion can be produced as a rare spontaneous stochastic event, as a consequence of mutations in PrP^C^-encoding gene, or induced by exogenous PrP^Sc^ that forces PrP^C^ misfolding, acting as a template [23,24,25]. Although still not unanimously accepted, this modality of structural alteration is not unique to PrP, but could explain certain self-propagating property of other amyloidogenic proteins associated with different neurodegenerative PCDs of CNS [26,27]. Similarly to all the other proteinopathy-associated neurodegenerative diseases, the emerging theory about the molecular mechanisms of neurotoxicity during TSEs postulates that the process of protein misfolding initiates a path of aggregation that goes through the formation of toxic soluble oligomers responsible of the neuronal degeneration and neuroinflammation [28,29]. The newly acquired physico-chemical properties of the oligomers are the most accredited determinants of their gain-of-toxicity; in particular, the increased hydrophobicity and resistance to proteolysis of the oligomers favors their interaction with cell structures and their propensity to aggregate in the neurons, altering cytoplasmic homeostasis [30,31,32]. In support of this hypothesis, it was demonstrated that neuronal death in all neurodegenerative diseases is associated with the generation of soluble misfolded protein oligomers. Remarkably, the structural refolding of amyloidogenic proteins, even unrelated to disease development, can initiate a similar aggregation process that generates soluble oligomers endowed with neurotoxic properties [13,33]. However, there is relative uncertainty as to the neurotoxic signaling of misfolded oligomers and how they interact with neurons. Synaptic impairment is the most prominent initial alteration observed in proximity of Aβ oligomer deposits, and accounts for the first cognitive impairment in AD [34,35,36,37], although mitochondria and/or lysosome damage have also been described as early pathogens during AD [38,39] and are responsible for neuronal death in experimental models of TSEs [32,40,41,42]. The possibility of non-specific interactions of oligomers with cell structures is suggested by the observation that protein misfolding often produces the exposure of hydrophobic residues that are otherwise buried within the native protein spatial structure, and the degree of hydrophobicity correlates with cytotoxicity [30,31,43,44]. Finally, neuronal damage can also be amplified by the sustained neuroinflammatory response triggered by oligomers’ direct activation of astrocytes and microglia [45,46,47,48,49,50,51]. Therefore, the relative contribution between the intracellular formation and the extracellular deposition of misfolded oligomers in determining neuronal death is unclear. The prominent localization of the aggregates can also vary according to the different diseases and, perhaps, the level of aggregation. While in PD and HD mutant α-synuclein and huntingtin are mainly recovered as intracellular aggregates, in AD and TSEs Aβ peptides and PrP^Sc^ are localized in extracellular plaques, although intracellular clusters within the endosomal/lysosomal vesicles have also been described. It has therefore been hypothesized that mature amyloid fibrils and their organization in plaques represent a defensive attempt to remove otherwise neurotoxic soluble oligomers. However, plaques are not just deposits of irreversibly inactivated peptides, but can undergo a continuous dynamic balance of entrapment and release of neurotoxic entities. Thus, amyloidogenic peptides may form aggregates containing insoluble fractions that dynamically cycle with the soluble fraction, which is able to travel from the extracellular space to the cytoplasm. The balance between the accumulation of oligomers in extracellular and intracellular pools of amyloids is derived not only from the dynamic equilibrium between the production inside the cells and their subsequent release, but also from the ability of neuron and glia to take up misfolded peptides from the extracellular space [52,53].

Following the amyloid theory and its more recent developments, world-wide research has been oriented towards developing therapeutic strategies against AD, as well as other PCDs, having as a shared rationale the removal of pathogenic oligomers. To this end, passive, rather than active immunotherapy, is at present under active investigation, and various monoclonal antibodies raised against oligomeric forms of Aβ peptides are currently in phase I and III clinical trials [18], although the initial results are not really promising [54,55]. On the other hand, the pharmacological restoration of cytoplasmic proteostasis is attracting growing interest, as it may represent a “physiological” strategy for preventing the accumulation of and/or removal of toxic oligomers, regardless of the nature of the misfolded proteins or the causes/mechanisms of their misfolding [11,56].

The process of autophagy, the major subject of the present review, can be pharmacologically induced in cellular and animal models of PCDs to induce neuroprotection. In the context of drug repositioning [57,58], a growing number of old and novel drugs, so far placed in human therapy to treat a wide number of pathologies, often unrelated to neurodegeneration, are currently under re-evaluation as autophagy inducers to be introduced as disease-modifying strategies against PCDs of CNS.

## 4. Cytoplasmic Homeostasis and Neuronal Survival: Loss of Autophagy Efficiency is Associated with Protein Aggregation and Neuronal Loss

Protein quality control in the cells is accomplished by two major, highly interconnected, proteolytic systems: UPS and autophagy [16]. It is estimated that almost 30% of de novo synthesized proteins become substrates for the proteolytic machinery upon covalent binding with ubiquitin. While proteasome- and CMA-dependent degradation targets specific proteins in their monomeric state, the elimination of aggregated protein is mediated by aggrephagy, a specific form of macroautophagy. Given the fundamental role of proteostatic mechanisms in ensuring viability in long-living and post-mitotic cells, it is not surprising that impairment of UPS or autophagy is associated with cognitive decline and neuronal death. In particular, AD, PD, ALS, polyglutaminopathies, and other less common forms of CNS amyloidosis display aggregation of misfolded proteins that can be found as extracellular deposits or intracellular inclusion bodies and aggresomes [59,60]. It should be made clear, however, that the presence of increased autophagosomes in association with misfolded aggregates is not proof of the consequentiality of these conditions, since it does not distinguish whether autophagy imbalance (i.e., increased autophagosome content) is the cause or the effect of protein aggregation. The post-mitotic nature of neurons determines that, along with aging, these cells accumulate sequential damage derived from life-spanning insults [61]. Mitochondria and lysosomes are the organelles that most strikingly develop consequences of aging, with the former increasing in number but decreasing in efficiency with respect to electron chain transport, and the latter being filled with indigestible lipofuscin that impairs their functioning [62,63]. Generally speaking, during the aging process, a progressive reduction of the cellular systems that ensure the maintenance of proteostasis occurs. Specific protein degradation by UPS and CMA is most affected, but macroautophagy, which should function as a *salvage* bulk cytoplasmic recycler, also becomes less efficient; it has been proposed that intralysosomal lipofuscin burden inhibits their fusion with autophagosomes, reducing autophagy efficiency [64].

AD, the most frequent neurodegenerative disorder, is characterized by extracellular deposition of amyloid plaques and intracellular tangles, composed by aggregated Aβ peptides and hyperphosphorylated protein tau [65], paralleled by a robust activation of lysosomal-mediated proteolysis, albeit displaying defects in execution [38,66,67]. Importantly, studies using cell lines and transgenic mice overexpressing Aβ precursor protein, or mutant forms of presenilin 1 (PS1), show that defective autophagy is an early feature in AD, causing a gradual but persistent accumulation of Aβ and hyperphosphorylated tau [15,66,68,69,70]. Post-mortem electron-microscopy and immunoblotting analysis of AD patient brains show that dystrophic neurons containing neurofibrillary tangles also evidence a high number autophagosomes filled with electron-dense material [71,72]; the amount of insoluble ubiquitinated proteins in conjunction with sequestrosome 1/p62 (SQSTM1/p62) is also significantly increased in AD patient brains when compared with age-matched controls, and positively correlates with the density of extracellular amyloid plaques [15,73,74]. High rates of autophagosome formation are also evident in primary cultures of neurons bearing AD-associated mutations, but, in contrast to wild-type controls, the former show defective proteolysis, similar to what is observed when autophagosome-lysosome fusion is pharmacologically hampered [15].

On these bases, it has been hypothesized that the extent of the age-dependent neuronal loss is directly correlated with the sharp decline in neuronal proteostasis efficiency, causing misfolded protein accumulation and neuronal death, whereas at younger ages this event is prevented by an efficient autophagy flux [61]. Interestingly, the impairment of autophagy-mediated proteostasis, which favors amyloid brain deposition and tau hyperphosphorylation [75], is associated with excessive and steady activation of the mammalian target of rapamycin (mTOR), which is abnormally elevated in AD patients [76].

PD, the second most common neurodegenerative disease, is characterized by the presence of dopaminergic dystrophic neurites in the *substantia nigra*, in which aggregation of ubiquitinated α-synuclein forms intracellular inclusion named Lewy bodies [77,78]. The most relevant cytoplasmic alterations that may possibly be involved in neuronal death are the accumulation of damaged mitochondria and of α-synuclein aggregates, resulting from exposure to still-undefined environmental toxins, or inherited defects in the enzymatic control of α-synuclein metabolism. Defective mitophagy and CMA have been observed in neuronal cultures bearing PD-associated mutations, in animal models of the disease, and in human PD-affected brains [79,80,81]. Genetic and pharmacological enhancement of autophagy has been able to reduce the amount of α-synuclein aggregates, oxidative damage and increase neuronal viability [82,83]. Analogously to AD and PD, autophagy defects have also been found in other proteinopathies, such as polyglutamine disorders and ALS, in which intraneuronal inclusions are associated with neuronal death [84,85,86].

Significant accumulation of autophagic bodies has been described, along with brain spongiform alterations, in TSEs [87]; in murine models of TSEs, in PrP^Sc^-infected cell lines, or in neuronal cultures treated with misfolded recombinant PrP fragments, an increased number of autophagosomes filled with electron-dense material containing PrP^Sc^ was reported. In these studies, the pharmacological activation of autophagy favored the clearance of the autophagosomes altogether, with reduced neurotoxicity. These data suggest that in the presence of intracellular PrP^Sc^ accumulation, neurons activate autophagy flux but are unable to complete the process [32,42,88,89]. Incomplete autophagy thus represents a prominent cause of neurotoxicity due to intracellular misfolded protein aggregation.

## 5. Potentiation of Autophagy as Neurodegenerative Disease Therapy

In the last two decades, several studies have reported the neuroprotective activity of rapamycin, one of the most powerful pro-autophagy agents, in both cellular and animal models of neurodegenerative diseases and, with some limitations, in humans [90]. In neuronal cultures, the toxicity of different amyloidogenic peptides can be counteracted by treatment with rapamycin [15,91,92]. Positive results have also been obtained in animal models bearing age-related, inherited, or neurotoxicity-induced neurodegeneration associated with protein misfolding [91]. The translation of these results into human therapy is highly desirable, since, apart from symptomatic pharmacological treatments, which significantly improve quality of life in PD patients and produce extremely limited cognitive improvement in AD patients, disease-modifying therapies, aimed at slowing down the cognitive and neurological decline in neurodegenerative disorders, are still lacking. Some encouraging results from properly structured clinical trials have been produced treating ALS-affected patients with lithium [93,94] and, more recently, a randomized, placebo-controlled, phase II clinical trial was started to evaluate the efficacy of rapamycin in patients affected by ALS [94]. As a common trait, all these approaches are intended to favor neuronal ability to remove cytoplasmic harmful entities, such as misfolded aggregation-prone proteins, or damaged mitochondria and lysosomes. Particular efforts have been focused on the possibility of repurposing known drugs in the neurodegenerative disease field, on the basis of their ability to stimulate macroautophagy in neurons. This pharmacological approach, distinct from the so-far ineffective current disease-modifying therapies, targeting a common step of neurotoxicity of all CNS degenerative conditions, regardless of the nature of the proteins that are associated with the different diseases, may represent a general therapeutic approach for PCDs, independently from etiology or the specific misfolded protein.

## 6. Molecular Control of Autophagy

The modulation of autophagic flux involves a complex signaling pathway integration that couples the detection of environmental alterations with functional cell responses, including proliferation, differentiation and, more generally, the maintenance of cytoplasmic homeostasis.

Mammalian target of rapamycin (mTOR) is a Ser/Thr kinase belonging to the family of phosphatidylinositol 3-kinase (PI3K)-related kinase (PIKKs), whose activity restrains constitutive autophagy at a low level when there is a shortage of nutrients or cell stresses, including organelles senescence/damage, and an increase of misfolded aggregation-prone proteins perturbs cytoplasmic homeostasis [95]. mTOR is the central partner of two hetero-oligomeric complexes called mTOR complex 1 (mTORC1) and 2 (mTORC2), which can be distinguished with respect to their protein composition, mechanisms of regulation and biological role. mTORC2 is involved in the prevention of apoptosis, control of glucose metabolism, and cytoskeletal plasticity, while mTORC1 controls cell cycle progression and the extent of autophagic flux in response to environmental changes.

In addition to mTOR, mTORC1 includes the regulatory associated protein of mTOR (RAPTOR), the mammalian lethal with SEC13 protein 8 (mLST8), the inhibitory subunit 40-kDa proline rich AKT substrate (PRAS40), and the DEP domain-containing mTOR interacting protein (DEPTOR) [96]. The best characterized pathway by which mTORC1 regulates autophagy is dependent on the activation of tyrosine kinase growth factor receptors. Their activation elicits class I PI3K activity, with subsequent conversion of phosphatidylinositol 4′,5′-biphosphate (PIP2) into phosphatidylinositol 3,4,5-triphosphate (PIP3). PIP3 recruits the Ser/Thr kinase AKT to the plasmamembrane, where it is phosphorylated and activated by two other kinases (PDK 1 and 2). In turn, AKT phosphorylates and inactivates the tuberous sclerosis complex (TSC) and PRAS40, preventing their inhibitory activity on mTOR [97,98]. Non-phosphorylated TSC2 represses mTOR kinase via the enhancement of GTP hydrolysis to GDP, in the small GTP-binding protein RAS homolog enriched in brain (Rheb), which is expressed on lysosomal membranes; as another small GTPase, Rheb cycles from an inactive GDP-bound to active GTP-bound form that activates mTOR kinase. In addition, PRAS40 directly interacts with and inhibits mTOR. AKT thus induces mTOR activity, blocking the inhibitory pathways regulated by TSC2 and PRAS40 [99]. Thus, in conditions of sufficient availability of trophic factors and energetic supply, a sustained stimulation of the PI3K-AKT pathway will result in the activation of mTOR via the continuous blockade of its inhibitors TSC2 and PRAS40. mTOR kinase sustains the phosphorylation of its downstream effectors p70 ribosomal protein S6 kinase 1 (S6K1) and eukaryotic translation initiation factor 4E (eIF4E)-binding protein 1 (4E-BP1) to promote nucleotide synthesis, ribosome biogenesis, protein synthesis, and cell proliferation. Conversely, trophic factor shortage reduces AKT activity allowing the inhibitory functions of TSC2 and PRAS40, which respectively reduces the amount of GTP-bound Rheb and binds to mTORC1 complex, inhibiting the phosphorylation of mTOR substrates, resulting in the activation of autophagy flux. Another major environmental stress that downregulates energy consumption in cells and stimulates autophagy is the reduction of glucose availability and the consequent increase of ADP/ATP ratio. The increase of AMP and ADP stimulates the activation of AMP-activated protein kinase (AMPK), which blocks mTORC1 activity through direct activation of TSC2 and inhibition of RAPTOR. Moreover, AMPK promotes autophagy downstream of mTORC1 by direct activation of phagophore-forming enzymatic complex unc-51-like kinase (Ulk1)1/2 (UKC), which is considered the initiator of the autophagic cascade. These conditions are the metabolic basis for autophagy activation, and TSC2 is now regarded to be a central station in which environmental nutrient availability or stresses are integrated.

Within the mTORC1 complex, RAPTOR acts as a crucial scaffold unit, allowing mTOR to bind and activate S6K1 and 4EBP1, when PRAS40 is phosphorylated by AKT. Importantly, RAPTOR is also the target of the autophagy inducers sirolimus (rapamycin) and tacrolimus (FK506); these antibiotics interact with a regulatory protein that drives them to RAPTOR to destabilize RAPTOR-dependent downstream mTOR signaling. Rapamycin, in addition to suppressing mTORC1 kinase activity, also induces the dephosphorylation of AMBRA 1, which precedes the activation of ULK and phagophore-forming enzymatic complex UKC and the class III phosphatidylinositol 3-phosphate kinase, both representing the initial steps of autophagy [100,101].

## 7. Autophagic Structures and Associated Protein Markers

The first autophagic structure to be formed is a cup-shaped double-membrane vesicle, called an isolation membrane or phagophore. According to the analysis of the lipid and protein content, phagophores stem from endoplasmic reticulum, but, for their expansion, receive further contributions from multiple sources, including recycling endosomes, mitochondria, Golgi and lipid droplets [102,103,104,105,106,107]. The entire process of nucleation, expansion, closure and, eventually, fusion with lysosomes is made possible by the recruitment of a wide array of proteins from cytosol. Among these factors, a mammalian orthologue of yeast Atg8, the microtubule-associated protein 1 light chain 3 B (LC3), drives the elongation phase of phagophores [108]. As described in a seminal paper by Kabeya and Coll., the 30 kDa full-length precursor pro-LC3 is cleaved at the C-terminus, forming a mature 18 kDa cytosolic form (LC3-I) that is further trimmed to originate a 16 kDa fragment (LC3-II), bound to phosphoethanolamine and inserted into the membrane of the elongating phagophore [109]. LC3-II not only allows phagophore elongation, but is also involved in the caging of aggregated proteins into phagosomes; its amino acidic sequence contains a binding region for the autophagy receptors SQSTM1/p62 and NBR1 (neighbor of BRCA1 gene1) that, in turn, recognize committed-to-digestion ubiquitinated proteins. Thus, during the elongation phase, LC3-II binds to ubiquitinated cytoplasmic material, favoring the accumulation of aggregated proteins within the phagophore [108,110,111]. The edges of phagophores eventually seal to form double-membrane vesicles, the autophagosomes [112,113]. The fate of newly formed autophagosomes is not uniquely directed towards fusion with lysosomes, but other vesicular entities can converge within the autophagic pathway. Endosomes, in particular, and multivesicular bodies can fuse with autophagosomes, before the arrival of lysosomes, resulting in the formation of amphisomes [6].

In the late stages of autophagy, autophagosomes travel along microtubules in the direction of the microtubule organizing center, where they interact with lysosomes, allowing the degradation of the cargo; of note is that the complete fusion with lysosomes, although often described, is not an absolute requirement for the formation of autolysosomes, and the passage of hydrolytic enzymes may also occur through transient or partial fusion among autophagosomes and lysosomes [114,115]. The fusion produces a net reduction in LC3-II content, since the fraction bound with the internal membrane of the autophagosome is digested together with the internal cargo. Accordingly, the amount of LC3-II is the most reliable marker of the number of autophagosomes in the cytoplasm. The final stage of autophagy produces the degradation of cargo macromolecules up to their constitutive elements and their release to the cytosol for recycling. Although representing a major goal of the entire autophagic process in mammals, nutrient recycling has been far less investigated and characterized than the preceding steps. Most knowledge of this process is derived from studies in yeasts, in which autophagosomes eventually fuse their outer membrane with acidic vacuoles, where they are digested along with their cargo, mainly through the activity of vacuolar proteinases [116]. Yeast vacuole membranes contain several proteins that have been associated with catalysis of autophagic content [117,118] and amino acid efflux to the cytosol [119,120,121]. The best characterized among these mechanisms of extrusion from vacuole are represented by integral membrane proteins, called autophagic vacuole transporter (AVT), which produce cytosolic efflux of amino acids in a relative selective manner.

## 8. Autophagy of Damaged Mitochondria and Lysosomes

The major cause of oxidative damage in aging cells or caused by exposure to environmental stresses arises from malfunctioning mitochondria in which the respiratory chain produces excessive amounts of reactive oxygen species (ROS), or when the antioxidant ability declines. This is particularly troublesome in neurons in which the energy demand requires numerous and hyperactive mitochondria. Renewal of mitochondria and mitochondrial protein is thus a relevant neuroprotective activity and can be accomplished by either proteasome activity or autophagy, but it is reduced during aging or under neurodegenerative conditions [79]. Of note, removal of most mild dysfunctional and oxidized proteins that are exposed on the outer mitochondria membrane can be achieved through a proteasome-mediated process of fixation that does not require the digestion of the entire organelle [122]. However, when the production of ROS exceeds a threshold level, it can impair the activity of proteasome itself, making macroautophagy an absolute requirement for removing damaged mitochondria and preventing cell death. In the latter case, a mitochondrial-targeted form of macroautophagy, for this reason called mitophagy, takes place, in which mitochondria are labelled in order to be recognized by autophagic machinery. Impairment of mitochondrial electron transport chain by defective respiratory complex I and ROS-induced oxidative damage are the major contributors for dopaminergic neuron death in PD, not only in inherited forms, but also in those induced by chronic exposure to insecticides rotenone and paraquat [123,124,125]. The best characterization of mitophagic mechanisms arises from studies on proteostasis defects associated with inherited forms of PD [126], where point mutations in pathogenic genes cause the impairment of this pathway. A key protein in mitochondrial commitment to degradation is the PTEN-induced putative protein kinase 1 (PINK1). PINK1 undergoes a rapid turn-over on the mitochondrial surface in healthy conditions, but in PD it accumulates on the outer membrane of depolarized or damaged mitochondria [127]. PINK1 recruits a cytosolic E3-ubiquitin ligase, named Parkin, that attaches ubiquitin chains to mitochondrial outer membrane proteins. Ubiquitinated mitochondria are subjected to proteasome-mediated fixation or recognized as degradable cargo by the interaction with SQSTM1/p62, and included within the phagophore [128]. Reduction of mitophagy efficiency is also part of the physiological alterations during normal aging. In these conditions, the increase of defective mitochondria cause overproduction of ROS and oxidative damage of lysosomal enzymes, followed by the accumulation of lipofuscins within the lysosomes [63,80,129]. Defects in mitophagy machinery are the main pathogenetic alterations in early-onset inherited parkinsonisms, in which the most relevant mutations occur in PINK1 and Parkin genes [79,80,81,130]. Similarly, damage in mitophagy has also been hypothesized to represent a risk factor in sporadic forms of other neurodegenerative conditions such as AD, ALS, and HD [131]. Rapamycin reduces rotenone neurotoxicity, stimulating the autophagic clearance of swollen mitochondria [112,132]. This evidence suggests that the impairment of mitochondrial-targeted autophagy results in predisposition to dopaminergic loss in the *substantia nigra* in these forms of PD.

Another source of potentially deadly derangement from cytoplasmic integrity is represented by malfunctioning lysosomes. Lysosomes’ crucial role is to ensure continuous quality control in protein and organelle turnover, thus requiring the perfectly calibrated selective permeability of their membrane. Massive rupture of the lysosomal membrane typically occurs after strong physico-chemical insults and produces rapid cell necrosis and significant inflammatory response in the surrounding tissue. In contrast, a subtler lysosomal membrane permeabilization is associated with apoptosis and has been described to result from a great variety of physiologically frequent stresses, including phospholipid oxidation by free radicals, exposure to lysosomotropic agents, lysosomal rupture by proteolysis-resistant bacteria or viruses and, importantly, accumulation of aggregated indigestible proteins. The limited diffusion of hydrolytic enzymes is a well-characterized stimulus for mitochondrial depolarization, followed by release of Cytochrome C, the formation of apoptosome and the execution of extrinsic apoptotic pathway. Clinical and experimental evidence suggests that lysosomal loss of selective impermeability in neurons occurs in physiological aging, and, possibly, contributes to neuronal loss in neurodegenerative diseases [133,134].

A recently identified pattern of cell response to lysosomal alteration, named endo-lysosomal damage response, is a defensive system executed by cells to (i) achieve recovery of lysosomal proteins from light damage, (ii) modify lysosomal production to compensate for loss of proteolytic competence, or (iii) remove irreparably damaged lysosomes. The latter point is achieved through activating a specific form of macroautophagy, similar for many aspects to mitophagy, which has been named lysophagy [135]. Lysophagy plays also a relevant role in antibacterial cell defense [136]. Altered lysosomes are recognized by galectins (cytosolic glycan-binding proteins) that function as ELDR sensors through the binding to β-galactosides in the inner face of the damaged lysosomal membrane. Target-specific forms of lysophagy have been described on the basis of the damaging agent, in which lysosomes recruit different kinds of galectins (Gal1, 3, 8, 9) to activate different downstream signals [136,137,138]. Overall, ubiquitinated proteins on lysosomal surface are recognized by autophagy receptors, among which SQSTM1/p62 drives the nucleation of the phagophore.

Remarkably, for the purposes of this review, there is growing evidence that disturbances of lysophagy make a relevant contribution to neuronal death in genetic forms of tauopathies and ALS, although it is still unclear whether neuronal loss is dependent on specific impairment of lysophagy or it results from a general disturbance of autophagy [137].

## 9. Aggrephagy is a Form of Macroautophagy Directed Against Misfolded Proteins

The ability to form multimeric complexes is an inherent feature of all proteins and is required for their biological functions. Thus, its correct execution is tightly regulated. Altered amino acid sequences caused by gene mutations or ribosomal malfunctioning, oxidative stress, and other processes can produce failures in the proper protein assembly, commonly referred to as “misfolding”. The generation of these aberrant proteins is often harmful to cells, because they might lose activity or gain cytotoxic properties. Many neurodegenerative disorders of CNS are associated with the accumulation of highly expressed neuronal proteins, whose misfolding causes exposure of hydrophobic groups to the cytosol, initiating a process of aggregation that ultimately produces insoluble clumps as extracellular or intracellular deposits [33,139,140].

In contrast to microautophagy and CMA, macroautophagy has been classically described as a non-specific system for bulk degradation of cytosolic proteins. Although this concept may still be appropriated to identify forms of autophagy ensuring recycling of macromolecule for cell survival to trophic stresses, a significant heterogeneity in intracellular targets, mechanisms of regulation, and biological meaning has recently been identified. In 2007, Overbye and Coll. introduced the term “aggrephagy” to describe autophagosomes engulfed by high levels of aggregated proteins and marked by the chaperonine Hsc70 (heat shock complex 70) [141]. Aggrephagy is a particular form of macroautophagy that allows the removal of aggregated proteins and contributes to determining neuronal sensitivity to alterations of proteostasis [10,142]. When needed, monomeric forms of misfolded or overexpressed proteins are digested by heat shock-mediated proteolysis or by UPS, which represent the most active cellular proteolytic systems under physiological conditions. In the absence of a proper activity of proteosomal proteolytic digestion, aggregation-prone proteins undergo a process of multimerization that eventually produces cytosolic aggregates that move along microtubules and concentrate at the microtubule organizing center (MTOC) to form large clumps called aggresomes [143,144]. The relevance of aggresome formation for cell survival is still a matter of debate, and it is unclear whether they represent solely inert end-points of a self-defense strategy of the cells, or if their removal is needed to prevent cell death. Since Hsc70 recognizes ubiquitinated proteins, it is conceivable that autophagosomes may act as scavengers that recognize and engulf specific proteins that have undergone ubiquitin-mediated modifications. This aggrephagy process involves receptors for cargos and adaptor proteins, such as SQSTM1/p62 and NBRI, which contain both a domain that recognizes the autophagic core protein LC3, and a site of interaction with ubiquitin residues on cargo proteins; they specifically select and direct aggregated proteins into the elongating phagophore that grows around the aggregate [145]. SQSTM1/p62, beyond recognizing poly ubiquitins marking misfolded proteins, has the ability to favor the formation of larger aggregates that are more easily recognized by the phagophore core machinery [110]. Another protein crucial for aggrephagy is the scaffold protein PI3P-binding autophagy-linked FYVE domain protein (Alfy), which stabilizes the complex between SQSTM1/p62 and the ubiquitinated proteins, allowing the recognition by the autophagic core complex composed by Atg5, Atg12, Atg16, and LC3 [146,147]. Alfy belongs to the so-called “autophagy adaptor molecules” and, in non-stressful conditions, is detected in both the nuclear membrane and inside the nucleus. However, a nuclear-cytoplasmic shift of Alfy localization occurs when neurons necessitate the elimination of aggregated proteins. Importantly, the presence of Alfy excludes non-ubiquitinated protein from autophagosomes, increasing the specificity of aggrephagy toward aggregated proteins only. Alfy activity primarily occurs in the brain, where aggrephagy is far more important [147] and is necessary to prevent neurotoxicity in animal models [145]. Aggrephagy then proceeds, analogously to classical macroautophagy, with the fusion between autophagosome and lysosomes and the degradation of SQSTM1/p62/NBR1 complex along with the cargo proteins [110,111,148]. In contrast to starvation-induced autophagy, the mechanisms of regulation of aggrephagy are less characterized and do not depend on the blockade of mTORC1. Instead, cytosolic migration of SQSTM1/p62 and Alfy from the nuclear membrane and the binding with target proteins represents an initiating event and, perhaps, a rate-limiting step in aggrephagy.

## 10. Pharmacological Control of Autophagy

**Figure 1:**
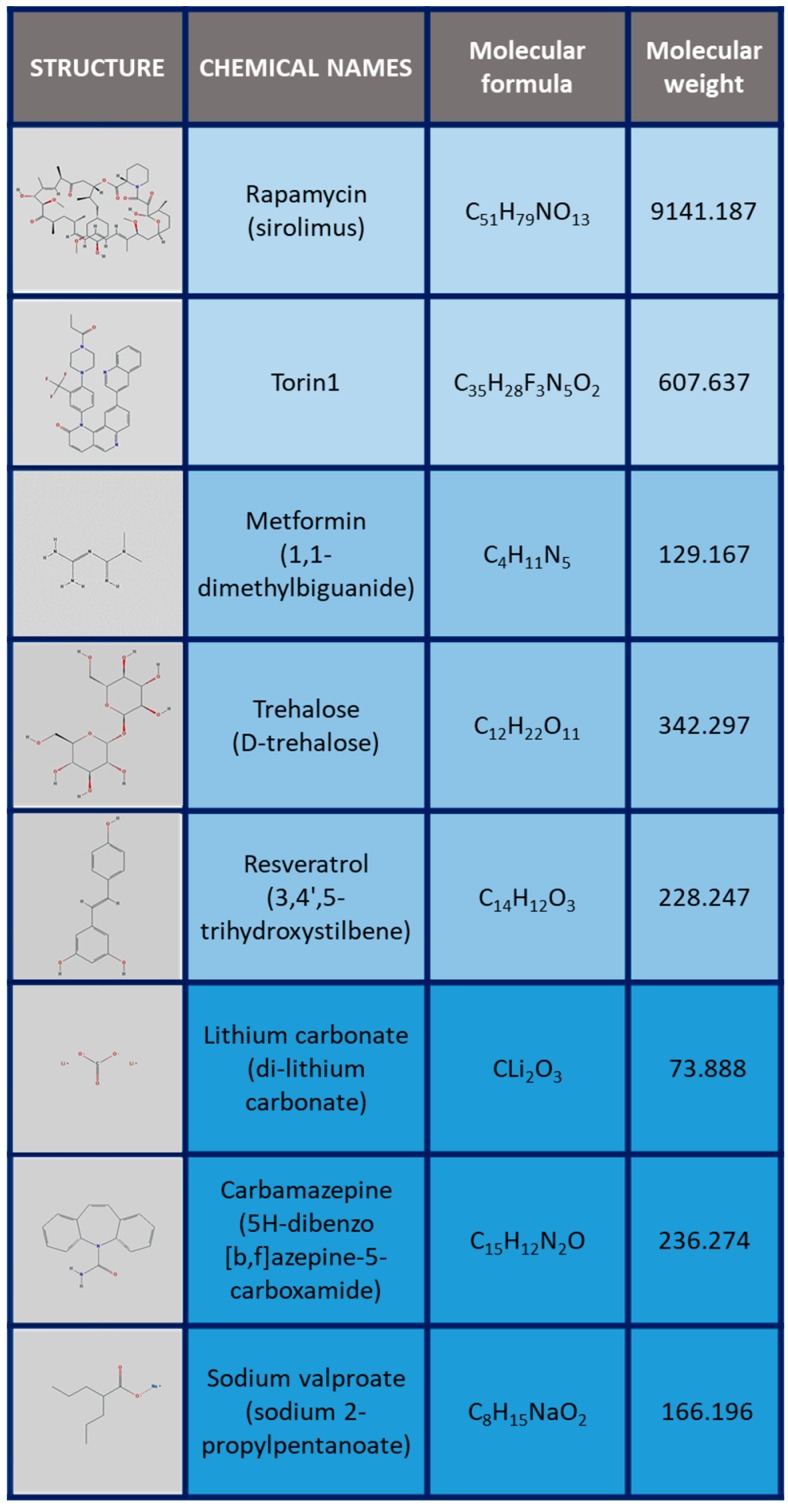
Structures and molecular weights of the main drugs acting as autophagy inductors.

**Figure 2:**
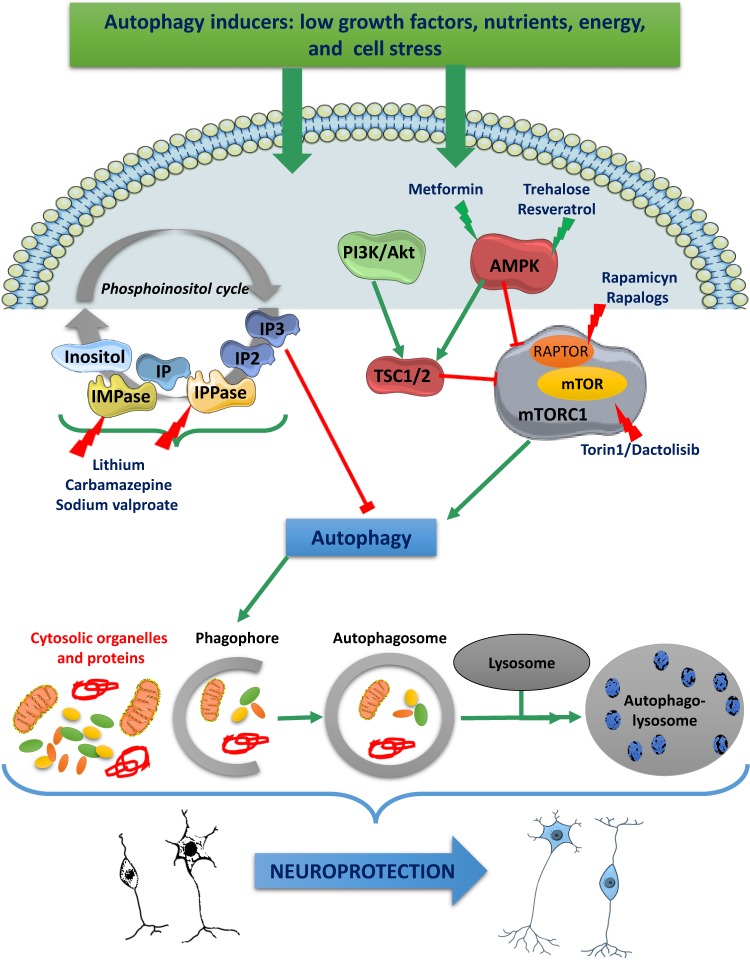

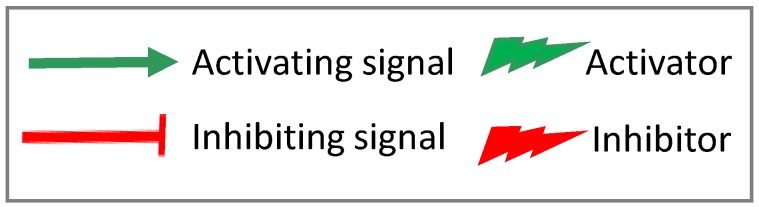
Overview of the main pharmacological strategies for inducing autophagy through targeting of mTOR-dependent, mTOR-independent and phospho-inositol-signaling pathways.

The role of autophagy as a pivotal regulatory mechanism of cell fate in aging, neurodegeneration, and carcinogenicity, along with its therapeutic potentiality, has stimulated significant efforts in the identification of autophagy-enhancing activity in drugs already used in human therapy, or directly developed for this purpose. The structures of the main drugs tested as autophagy activators discussed in the next paragraphs are depicted in Figure 1, while their corresponding molecular targets are reported in Figure 2.

Autophagy stimulation pathways converge to regulate mTOR. The growth factor pathway comprises PI3K/Akt/TSC/mTOR signaling and the ATP-energy depletion-activated AMPK/TSC/mTOR cascade, leading to TORC1 inhibition and autophagy induction.

Rapamycin and rapalogues enhance autophagy by stabilizing raptor-mTOR association and inhibiting mTOR activation; mTOR inhibition could also be achieved with torin1 and dactolisib.

Agents activating AMPK, such as metformin and trehalose or resveratrol, trigger AMPK-dependent mTOR inactivation.

One of the mTOR-independent autophagy activation pathways is a cascade of reactions triggered by low intracellular levels of inositol. This cascade is activated by the block of G-protein coupled receptors, leading to a reduction of IP3. Inositol arises as a result of hydrolysis of two phosphate moieties from IP3, forming IP2 and IP, IP is subsequently hydrolyzed IMPase.

Autophagy can be induced by drugs that decrease inositol or inositol IP3, such as lithium by IMPase, and carbamazepine and sodium valproate via impairment of inositol synthesis.

Abbreviations: mammalian target of (mTOR), mTOR complex 1 (mTORC1), regulatory-associated protein of mTOR (RAPTOR), tuberous sclerosis protein 1/2 (TSC1/2), phosphatidylinositol-4,5-bisphosphate 3-kinase (PI3K), protein kinase B (AKT), 5’AMP-activated protein kinase (AMPK), inositol triphosphate (IP3), inositol monophosphatase (IMPase), inositol polyphosphatase (IPP).

### 10.1. Rapamycin and Rapalogues

The blockade of mTORC1 activity, physiologically occurring during nutrient shortage [149], can be pharmacologically achieved by two structurally related macrolide antibiotics, sirolimus (rapamycin) and tacrolimus (FK506), and their derivatives (rapalogues) everolimus and temsirolimus; these drugs represent the most effective and powerful autophagy activators so far. Studies on brain tumors have demonstrated that rapamycin and its derivatives are able to cross the blood-brain barrier, with the newer compounds, such as temsirolimus, being more effective in this regard [150,151].

Rapamycin and tacrolimus reduce the kinase activity within mTORC1 upon binding with the intracellular receptor FK-506-binding protein 12 (FBP_12_), which, in turn, recognizes a binding site on mTOR. The complex FBP12-mTOR counteracts the scaffolding property of RAPTOR, preventing the mTOR dimerization and activation [152]. Rapamycin, tacrolimus and the rapalogue everolimus, have been introduced in therapy as immunosuppressant drugs to avoid transplant rejection; later on, everolimus became a first choice treatment for long-term control of tuberous sclerosis-associated angiomyolipomas [153,154] and for the therapy of neuroendocrine tumors of the gastro-entero-pancreatic tract [155].

The ability of rapamycin to reduce protein synthesis and block cell cycle progression produces immunosuppressive, antifungal, and anticancer activity; furthermore, rapamycin-dependent mTORC1 inhibition has recently been reported to function as a potent activator of autophagy. There is substantial evidence that the activation of autophagy flux induced by all rapamycin analogues provides neuroprotective effects in experimental models of neurodegenerative diseases, preventing the accumulation of aggregation-prone proteins and increasing neuronal viability [92,156]. The ability of rapamycin to restore autophagy flux has also been demonstrated in an in vitro TSE model of neurotoxicity in which neuronal death was induced by the recombinant misfolded PrP fragment 90–231. In this model, PrP90–231 rapidly accumulates in aggregates within neurons which try to remove them by activating the autophagy pathway. However, autophagic flux cannot be completed, and cells die by apoptosis, displaying a great number of unresolved autophagosomes [42]. In the presence of the autophagy-enhancing activity of rapamycin, the autophagic flux was completed and neurons survived. Similarly, tacrolimus also stimulates a sustained autophagic activity, leading to the degradation of newly misfolded PrP^Sc^ molecules [157]. The efficacy of rapamycin as an inducer of autophagy is unquestionable, but the benefits of its chronic administration in a clinical setting are significantly hampered by undeniable and common adverse reactions, including infections of the respiratory and urinary tracts, gastrointestinal pain, thrombocytopenia, and dyslipidemia [94]. Nevertheless, rapalogues, frequently prescribed as anti-cancer agents, are currently also under investigation for their ability to reduce the severity of neuronal loss due to proteinopathies, in view of their being a potential therapeutic extension to long-term treatment of neurodegenerative diseases [90].

Temsirolimus, an esterified rapamycin derivative, displays a remarkable antiproliferative activity in several forms of solid or hematological tumors, and it has recently been approved for treatment of advanced renal cancer [158]. Importantly, temsirolimus displays more tolerable side-effects than rapamycin and has no immunosuppressant activity. Temsirolimus demonstrated a remarkable capacity to stimulate autophagy and prevent neuronal death in vitro as well as in animal models of neurodegenerative proteinopathies induced by polyglutamine expansion of huntingtin [84] or ataxin-3 [159]. In transgenic mice bearing amyloid precursor protein (APP) overexpression, and point mutations of PS1 and tau genes, models of familiar AD, temsirolimus contrasted brain deposition of insoluble aggregates of Aβ and phosphorylated tau, lowering the amount of neurofibrillary tangle and apoptotic neurons. Importantly, temsirolimus-treated mice also exhibited better cognitive and motor performances in comparison with age-matched, untreated mutants [160,161,162]. Recently, significant beneficial activity of temsirolimus has also been observed in MPTP-induced animal models of PD: motor performance deficits, loss of dopaminergic neurons, and accumulation of α-synuclein were all prevented by simultaneous treatment with temsirolimus [163].

Finally, everolimus also showed a promising neuroprotective activity in AD animal models since, after acute intrathecal administration, reduced brain content of Aβ and hyperphosphorylated tau, via mTOR inhibition, and delayed animal cognitive decline were observed [164].

These encouraging results in support of mTOR inhibitors as pro-autophagic agents currently favors a great number of preclinical studies on innovative drugs that, although mainly focused on oncology, might expand the horizons of autophagy-based neuroprotective therapy [90].

### 10.2. ATP Analogues

ATP analogues can be sub-grouped, on the basis of their pharmacodynamics, as dual mTOR/PI3K inhibitors and pan-mTOR inhibitors [165].

Compared to rapamycin, dual mTOR/PI3K inhibitors have the advantage of producing the block of both TORC1 and TORC2 complexes. Among these, dactolisib (NVPBEZ235), developed as an antitumor drug, is able to reduce the in vitro toxicity of Aβ1-42 in mouse hippocampus, and to reverse the subsequent memory impairment [166]. Pan-mTOR inhibitors, showing better antitumor properties than rapamycin, have also been studied for neuroprotective activity. Among these, an interesting novel compound, although pharmacokinetically limited, is Torin1 which is able to inhibit the kinase domain of both TORC complexes in a highly specific manner, without appreciable effects on PI3K [167]. Recently, Torin1 demonstrated anti-aging capacity as a dietary supplement in a fly model without altering other parameters that may influence lifespan [168].

### 10.3. AMPK Activators

Metformin, the first line drug for type II diabetes, whose long-lasting practice in human therapy has evidenced excellent tolerability, is now attracting significant interest as an anti-aging, anti-ischemic and anti-cancer agent. Chronic administration of metformin for hypoglycemic purposes has been reported to reduce the incidence of cancer, stroke, AD, and PD in diabetic patients, in which the incidence of these pathologies is otherwise greater than in the non-diabetic population [169,170,171,172,173,174]. Metformin pro-autophagic activity is mediated by the activation of the AMP-activated protein kinase (AMPK), which has been proposed to contribute to its antiproliferative activity [174]. Metformin acts inducing AMPK activity through direct LKB1-mediated phosphorylation or, indirectly, through the reduction of mitochondrial production of ATP [175,176]. Active AMPK stimulates the formation of autophagosomes by inhibiting mTORC1 or eliciting the direct activation of its downstream effector ULK1. [177,178,179]. The growing interest in AMPK as a regulator of autophagy and the neuroprotective potential of this process is currently situating metformin and other AMPK activators as novel drugs endowed with anti-aging or anti-PCDs activity. Importantly, there is evidence that induction of macroautophagy, with the consequent prevention of protein aggregation or accumulation of damaged mitochondria and lysosomes, is not a unique neuroprotective effect of AMPK-dependent mTORC1 inhibition. Indeed, AMPK also improves mitochondrial function and exerts anti-inflammatory and antiapoptotic activities [180]. The most promising future application of AMPK activators, and in particular of metformin, is the therapy of PD in which multiple downstream effectors of AMPK activation directly contribute to the prevention of *substantia nigra* dopaminergic neuron death [181]. Dopaminergic neurons of *substantia nigra* are particularly sensitive to the age-dependent decline of the antioxidant system activity and reduced mitochondrial efficiency. Hence, AMPK activators, shifting the energy balance toward reduction of ATP consumption, along with the enhanced mitophagy and prevention of α-synuclein aggregation, can act as possible PD-modifying drugs. Although no results from clinical trials with metformin in PD patients have so far been reported, there is preclinical evidence that AMPK activation in neurons is protective, and the treatment with metformin can ameliorate symptoms and pathology in animal models of toxin-induced PD [182,183]. In 2017, a pilot study on patients diagnosed with mild cognitive impairment or early AD showed that treatment with metformin improved executive function, learning and memory [184]. Although it is difficult to ascertain the contribution of autophagy to the reported cognitive improvement, these results, associated with the capacity of metformin to cross the blood-brain barrier and its well-tolerated profile in elderly patients, represent significant incentives for further investigations on metformin’s neuroprotective activity.

An intriguing novelty in the research into neuroprotective compounds was introduced by the description of the cardioprotective, antiaging and antioxidant properties of the natural polyphenol resveratrol [185]. It was demonstrated that resveratrol effect is, in part, mediated by calorie-restriction, mimicking activity and induction of autophagy through AMPK activation [186,187]. Recently, the therapeutic promise of resveratrol was realized in the demonstration that it may provide neuroprotection in cellular and mouse models of PCDs, including AD, HD, ALS and PD [180]. Of note, it has been demonstrated that the neuroprotection exerted by resveratrol is partly due to the stimulation of AMPK- and PARKIN-mediated mitophagy, suggesting the value of further exploration of its therapeutic potential, particularly in neurodegenerative conditions associated with mitochondrial impairment [180,188,189]. Interestingly, other AMPK activators, and metformin in particular, can also restore mitophagy-modulating PARKIN activity [180].

### 10.4. Inhibitors of Phosphoinositol Turnover

Lithium, a mood-stabilizing drug used in psychiatry since 1950 for the treatment of bipolar disorder, has recently demonstrated the ability to provide neuronal protection against several stresses, including traumatic injuries, excitotoxicity, environmental toxins, neuroinflammation and proteinopathies [190,191,192,193]. In particular, neuronal death, occurring after cytoplasmic aggregation of disease-associated proteins such as Aβ peptides, as well as mutant huntingtin, α-synuclein, or superoxide dismutase, is significantly reduced by lithium through the induction of autophagy [194,195,196]. In these studies, autophagy activation by lithium favors the removal of misfolded proteins along their oligomerization path, preventing the neurotoxicity of soluble oligomeric forms, and the formation of large cytosolic insoluble aggregates. The neuroprotective and pro-autophagic activity of lithium has also been reported in vivo, using mouse models of ALS and tauopathies [93,197]. In these mice, oral or parenteral chronic lithium administration delayed sensory and motor decline in comparison with untreated animals; brain histochemical analysis often showed lower amounts of SQSTM1/p62 in lithium-treated mouse brains, whereas autophagosome-bound LC3-II was significantly increased, indicating that lithium can improve the efficacy of autophagosome-mediated proteolysis [93,197]. Given the complex biochemical signaling activated by lithium, the definition of the effectors of its neuroprotective activity is still not completely clear, but it is likely that lithium pro-survival efficacy does not rely entirely on the activation of autophagy. Lithium, in its cationic monovalent form, can compete with magnesium divalent cation (Mg^2+^) to inhibit many enzymes that require magnesium as a cofactor [198]. Lithium causes the direct inhibition of inositol monophosphatase (IMPase) and inositol polyphosphatase-1 (IPP), blocking the phosphoinositide cycle and causing a net reduction of intracellular 1,4,5 inositol triphosphate (IP3). Interestingly, IP3 acts as autophagy suppressor through its binding to intracellular receptors located in the endoplasmic reticulum and mitochondria [199]. Another enzyme whose inhibition is likely to mediate the lithium neuroprotective effect is the glycogen synthase kinase-3 β (GSK-3β); the activity of this enzyme facilitates tau hyperphosphorylation and augments Aβ accumulation, playing a role in AD-related neurite dystrophy and neuronal death [200]. Chronic treatment with lithium is also beneficial through other mechanisms, including the expression of antiapoptotic proteins, the reduction of oxidative damage in mitochondria, the production of neurotrophic factors and the proliferation of neuronal progenitor cells [198]. Given all these necessary premises, however, it must be remarked that the impairment of inositol turnover is the most relevant trigger for lithium induction of autophagy [196,197]. The neuroprotective activity of lithium in cell cultures and in mouse models of AD provided the rationale for clinical trials in patients in the mild and moderate phases of the disease [198,201,202,203]. These trials mainly addressed the progression of cognitive impairment, and did not report a significant improvement of life expectancy of the enrolled patients, although lithium was well tolerated. Only a limited slowing-down of cognitive deterioration was observed in lithium-treated patients, associated with a significant reduction of hyperphosphorylated tau in cerebrospinal fluid [198,203]. Similar possible disease-modifying capacity of lithium was also considered for ALS. Lithium stimulated post-injury neurite sprouting, synaptogenesis and reduced ALS-like pathology progression in transgenic mice [204,205,206]. Regrettably, the translation of lithium potential in human therapy for ALS has produced, thus far, conflicting results. Distinct clinical trials that have been conducted with daily treatment with lithium for at least 12 months in ALS-affected patients have reported efficacy or no effects with respect to reducing the progression of disease and symptomatology, and increasing survival [207,208]. The potential therapeutic use of lithium has also been tested in HD and PD. It was reported that neuron death and accumulation of cytoplasmic aggregates of mutant forms of huntingtin and α-synuclein is significantly counteracted by lithium administration [194,196,209].

Carbamazepine (CBZ) and valproic acid (VPA), two antiepileptic and mood-stabilizing drugs, share with lithium the ability to inhibit the phosphoinositide cycle and neuroprotective potential, as observed in neuronal cultures and animal models of neurodegenerative disease [210,211,212]. Of note, both VPA and CBZ counteracted the accumulation of protein aggregates through the increase of autophagy [211,213,214]. However, in light of the preclinical and clinical data available so far, a definite evaluation of the utility of lithium and other mood-stabilizing drugs in neuroprotective therapy is premature and requires further investigation. In contrast to rapamycin, lithium, CBZ and VPA do not directly affect the enzymatic activity of mTORC1, and do not prevent the phosphorylation of the mTORC1 downstream effectors S6K1 and 4EBP1, thus displaying no immunosuppressant activity. Synergistic neuroprotective activity between lithium, VPA, and rapamycin has been reported in different animal models of inherited neurodegenerative pathologies [206,215] and represents a promising rationale for a deeper characterization of their pharmacodynamics as anti-PCD agents.

### 10.5. Trehalose

Trehalose is a natural disaccharide derived by the condensation of two molecules of glucose. It is particularly abundant in bacteria, yeast, insects, and other invertebrates, where it acts to protect cells against environmental stresses, particularly preventing protein denaturation [216,217,218]. The protein-stabilizing activity of trehalose is not limited to the organism in which it is produced, but can be observed when exogenously administered to mammalian cells, in which it protects protein integrity in freeze-drying procedures [219]. Trehalose also shows neuroprotective potential against aggregation-prone proteins, as demonstrated in cell-free systems, where it inhibits amyloid fibril formation from Aβ peptides, polyglutamine-containing myoglobin and insulin. Moreover, trehalose prevents neuron death induced by Aβ1-40 and mutant huntingtin, and reduces amyloid deposition and striatal atrophy in a mouse model of HD [220,221,222]. Given this neuroprotective potential of trehalose against PCDs, the characterization of its protein-stabilizing activity has attracted significant interest. Different mechanisms have been proposed to explain the anti-aggregating and neuroprotective effects of trehalose, including the direct binding to proteins, favoring the maintenance of a soluble state, and the inhibition of aggregation [222]. Trehalose exerts neuroprotection in cellular and animal models of PCDs caused by mutant forms of huntingtin, α-synuclein, tau, or SOD1, or by the inhibition proteosomal activity. In all these models, the reduction of neurodegeneration was associated with an increased number of autophagosomes, degradation of mutant proteins, and improved mitochondrial function [85,222,223,224,225]. Importantly, trehalose neuroprotection was reverted by the autophagy blocker 3-methyladenine, suggesting that macroautophagy plays a major role in trehalose activity [85]. However, since trehalose does not reduce the phosphorylation of the two main mTOR effectors, S6K1 and 4E-BP1, its activity is likely not dependent on the canonic rapamycin-mediated blockade of mTOR. Conversely, trehalose activity was proposed to be mediated by the activation of gene transcription for many autophagy-related and antioxidant proteins [226,227,228]. Moreover, studies on liver non-alcoholic fatty lipid disease demonstrate that trehalose causes AMPK-dependent activation of autophagy to determine the inhibition of glucose transporter at hepatocytes plasmamembrane [229,230]. Two important hints outline the potential of trehalose as neuroprotective agent in PCDs: (i) substantial evidence supports that trehalose stimulates the clearance of oligomers, the main neurotoxic entities, rather than the removal of large aggregates [231], (ii) trehalose stimulates autophagy independently of mTOR inhibition and its neuroprotective activity was additive with that of rapamycin [232]. In this regard, considering the potential toxicity of rapamycin and rapalogues, particularly in conditions of chronic treatment, the possibility of reducing the rapamycin dose through co-administration of a clearly tolerable molecule such as trehalose should definitely be pursued in the future.

### 10.6. Screenings for Novel Autophagy Enhancer Drugs

Potential novel pro-autophagic drugs were screened on the basis of their effect on cell proliferation, clearance of long-lived proteins, or on the expression of autophagy-related proteins. [233,234]. Sarkar and Coll. developed a yeast-based screening of substances able to strengthen the cytostatic activity of rapamycin, with the aim of identifying pro-autophagy effects on mammalian cells. In total, 21 molecules were detected and collectively grouped under the acronym “SMERs” (small-molecule enhancers of rapamycin) for their additive effect on antiproliferative activity of rapamycin. Three of these compounds were characterized in detail for their ability to accelerate mTOR-independent clearance of mutant α-synuclein and huntingtin in stably transfected cells. Notably, the neuroprotective activity of SMERs was successfully tested in vivo in a fly model of neurodegeneration induced by the expression of mutant huntingtin [85,233]. The same purpose was adopted to screen pro-autophagic activity of drugs already approved for human therapy, although with different indications from neuroprotection. Zhang and Coll. analyzed more than 30 known drugs for their capacity to elicit autophagy, and found that eight structurally unrelated compounds, mostly belonging to antipsychotic and calcium antagonist families, stimulated autophagosome formation and the degradation of aggregation-prone long-lived peptides [234]. In a contemporary, albeit independent, screening, other mTOR-independent activators of autophagy were identified, including, but not limited to, calcium antagonists, which could be effective in the intracellular clearance of aggregation-prone huntingtin and α-synuclein mutants [235]. Importantly, both studies provided proofs of principles of the neuroprotective repurposing of drugs already validated for prolonged therapy in humans. The most promising drugs, whose repositioning as pro-autophagic compounds is currently under investigation as neuroprotective agents, are listed in Table 1.

## 11. Future Development of Autophagy Enhancers in Neurodegeneration Therapy

The aging process produces an inevitable reduction of the efficiency of most physiological functions, representing a risk factor for the development of several neurodegenerative disorders, including AD and PD [168]. A key topic of research on aging has been, for a long time, the characterization of the cellular mechanisms that affect neuronal sensitivity to neurotoxic stresses, focusing on those whose decline in efficiency with aging allows intracellular accumulation of neurotoxic misfolded proteins. Of note, there is growing evidence that neuronal resistance to aging-related processes can be ameliorated by either nutritional or pharmacological strategies. The same treatments can also be successful in delaying the onset of sporadic and genetic neurodegenerative diseases [236,237]. Autophagy, through its renewing function of cytoplasmic content, which in neurons assumes crucial life-saving effects, is the main target of these novel approaches. Remarkably, autophagy works as a constitutive process that degrades bulk cytoplasm cargo under normal circumstances, or can be up-regulated under nutrient shortage when a rapid supply of nutrients is needed [149]. In addition to this non-specific recruitment of nutrients, autophagy can be specifically targeted to remove misfolded proteins during their path to aggregation or damaged organelles, thus acting as an inhibitor of cell death. This is particularly evident in neurons in which autophagy inhibition causes neuronal loss and intracellular accumulation of structurally altered proteins [238]. Moreover, the execution of apoptosis following neurotoxic insults can be prevented by contemporary activation of autophagy.

Protein quality controls, including proteasome-mediated degradation of short-lived proteins and all forms of autophagy, decline with aging, and this dysfunction favors the accumulation of damaged proteins and organelles in the cytoplasm [239,240]. To preface the possible placement of autophagy-enhancers as disease-modifying therapies against PCDs, it is necessary to note that, to date, all the clinical trials that have been performed with these compounds have produced, at most, limited improvement of cognitive scores, with no beneficial effects on survival [93,207].

The evidence that long-term non-pharmacological interventions, in particular caloric restriction or intermittent fasting, can significantly delay brain atrophy and cognitive decline [237], sharply contrasts with the failure of even the most promising pro-autophagic drugs in anti-PCD clinical trials.

It is unquestionable that the delay existing between therapy onset and actual neuronal damage in human neurodegenerative diseases can barely be modeled in animals. This discrepancy represents a major hindrance for the efficacy of disease-modifying therapies when translated from preclinical to clinical settings. For this reason, to optimize anti-aging therapies, it is recommended that the treatment begin well before the age “symptomatology” onset [241]. Of note, an important clinical trial was recently started for preventive purposes [242], enrolling still asymptomatic subjects that are at risk of developing a rare form of prion disease, fatal familial insomnia, since they bear a high-penetrance mutation of the *PRNP* gene [243]. Although this trial is still currently in progress, and it is not being performed using autophagy-enhancing molecules, its results could represent a future proof of concept regarding the opportunity to begin neuroprotective treatments in individuals at risk for neurodegenerative diseases at the very beginning of neurological onset, or even in the asymptomatic phase. Therefore, the decision to determine an early start of neuroprotective strategies should be based on: (i) an accurate diagnosis that could evaluate risk factors and evidence light symptoms as early as possible; and (ii) the availability of relatively side-effect-free drugs that patients, in early or still asymptomatic phases of disease, could tolerate for years if not for decades.

Rapamycin and rapalogues are, to date, the most powerful autophagy enhancer agents, although they are loaded with frequent and unwanted effects that currently limit their use to few selected conditions, such as anti-cancer and anti-transplant rejection therapies [244,245]. The recent great surge of scientific interest in autophagy as an anti-aging strategy stimulated the identification of molecular pathways that control the process and led to the definition of new targets for less toxic molecules to be used for long periods under non-life-threatening conditions. In this regard, the antipsychotic and antiepileptic drugs lithium, VPA and CBZ, which antagonize IP3 autophagy-repressing activity, represent proof-of-principle regarding the possibility of activating phagophore nucleation without blocking the kinase activity of mTOR [195]. Other molecules that have a well-established positioning in human therapy against a wide number of pathologies are currently under reevaluation by virtue of their promising, although still not-well-defined, pro-autophagic activity [85,233,235]. In addition, molecules so far devoid of clinical indications are able to activate mTOR-independent autophagy; among these, the disaccharide trehalose and grape polyphenol resveratrol convincingly show neuroprotective and pro-autophagic effects through multiple mechanisms, including the activation of transcription factors for autophagy-related proteins and antioxidants [226,227,228] or mimicking starvation-dependent reduction of glucose uptake and AMPK activation [229,230,246]. As already mentioned, all these substances have demonstrated anti-aggregating and neuroprotective properties, but have so far attracted limited interest towards translation into clinical research, as PCD disease-modifying drugs. In this regard, several clinical trials, reported in Table 2, are ongoing to test potential neuroprotective activity of autophagy enhancers, in the early phases of AD, HD and ALS, and these are expected to be concluded in the near future.

Evidence of successful additive autophagy activation by co-treatment with the mTOR-dependent rapamycin and mTOR-independent trehalose in a PD mouse model was recently reported [232], suggesting the efficacy of non-toxic low-dose, long-term combination treatments. It is also important to remark that the classic division of autophagy into micro, macro and chaperone-mediated has recently been challenged by clear evidence that macroautophagy does not just remove bulk cytoplasmic content; rather, neuroprotective macroautophagy is often specifically targeted to damaged organelles or proteinaceous aggregates. Hence, improved knowledge of such heterogeneity prefigures a futurible combined pharmacological approach in which the additive effects of two or more drugs might provide neuroprotection through specific action on multiple cell stresses that may be differently relevant in each PCDs.

## 12. Conclusions

The role of autophagy is becoming more and more relevant in different physiological and pathological conditions. In particular, several drugs acting through the modulation of the autophagic flux are now currently being used or are in clinical trials in oncology. However, the unifying theory regarding the molecular mechanisms of neuronal death in different neurodegenerative conditions in which defective autophagy leads to oligomers accumulation has prompted the analysis of the potential use of several pro-autophagic drugs also in these conditions. In particular, both novel and repositioned molecules are currently in preclinical and clinical trials to induce clearance of misfolded oligomers via the potentiation of the autophagic pathway.

## Figures and Tables

**Table 1:** Neuroprotective drugs with pro-autophagic properties.

Drug	Clinical Indications	Pro-Autophagic Mechanism	References
**mTOR inhibitors**	Anti-fungal, immunosuppressant, anti-cancer	Destabilization of mTOR-RAPTOR complex	[157,164,165]
Rapamycin (Sirolimus)			
Temsirolimus			
Tacrolimus			
Everolimus			
**ATP analogues**			
Torin 1/2	Anti-cancer	Blockade of ATP binding site on mTOR	[168]
Dactolisib			[166]
**Phosphoinositide cycle inhibitors**			
Lithium	Mood stabilizers	IP3 depletion, GSK-3β inhibition	[195,197,198,210]
Valproic acid	Anti-epileptic, mood stabilizers		
Carbamazepine	Anti-epileptic, mood stabilizers, analgesic		
**AMPK activators**			
Metformin	Antidiabetic	AMPK activation	[173,176]
Trehalose *	Antioxidant	GLUT blockade	[221,222,223,224,225,226,227,228,229,230,231]
Resveratrol *	Antioxidant	AMPK activation	[188,189]
**Ca^2+^ antagonists**			
Verapamil	Anti-arrhythmic and antihypertensive	IP3 depletion, calpain inhibition	[233,234,235]
Nimodipine			
Nitrendipine			

* unconventional.

**Table 2:** Ongoing clinical trials for PCDs using autophagy-enhancing drugs. Data were collected from NIH, US national Library of medicine (www.ClinicalTrials.gov) using “autophagy” and the drug names as key-words; only active trials are reported.

NCT Code	Drug Name	Disease	Stage of Disease	Purpose	Phase	Duration of Treatment	Estimated Study Completion Date
03185208	Lithium	AD	MCI *	Delay passage to overt dementia	4	2 years	March 2022
02336633	Resveratrol	HD	Early phase	Delay caudate atrophy	NA	1 year	Jan 2019
03359538	Rapamycin	ALS	all	Improvement of ALSFRS **	2	18 weeks	Apr 2019
03272503	Pimozide	ALS	all	Delay Progression	2	22 weeks	Dec 2019

* Mild cognitive impairment. ** Amyotrophic lateral sclerosis functional rating scale.

## Abbreviations

Alfy	Autophagy-linked FYVE-domain protein
AD	Alzheimer’s disease
AMPK	AMP-activated protein kinase
ALS	Amyotrophic lateral sclerosis
Aβ	β-amyloid
CBZ	Carbamazepine
PrP^C^	Cellular prion protein
CNS	Central nervous system
CMA	Chaperone-mediated autophagy
DEPTOR	DEP domain-containing mTOR interacting protein
4E-BP1	EIF4E-binding protein 1
eIF4E	Eukaryotic translation initiation factor 4E
GSK-3β	Glycogen synthase kinase-3 β
Hsc70	Heat shock complex 70
HD	Huntington’s disease
IMPase	Inositol monophosphatase
IPP	Inositol polyphosphatase-1
IP3	1,4,5 inositol triphosphate
mLST8	Mammalian lethal with SEC13 protein 8
mTOR	Mammalian target of rapamycin
LC3B	Microtubule-associated protein 1 light chain 3 B
MTOC	Microtubule organizing center
mTORC1	MTOR enzymatic complex 1
mTORC2	MTOR enzymatic complex 2
NBR1	Neighbor of BRCA1 gene1
PD	Parkinson’s disease
PIP2	Phosphatidylinositol 4′,5′-biphosphate
PIP3	Phosphatidylinositol 3,4,5-triphosphate
PI3K	Phosphoinositide 3-kinase
PIKKs	PI3K-related kinase
PrP^Sc^	Prion protein Scrapie
PRAS40	Proline rich AKT substrate 40-kDa
PCDs	Protein conformational disorders
PINK1	PTEN-induced putative protein kinase 1
Rheb	RAS homolog enriched in brain
RAPTOR	Regulatory associated protein of mTOR
ROS	Reactive oxygen species
S6K1	S6 kinase 1
SQSTM1/p62	Sequestrosome 1/p62
SMERs	Small-molecule enhancers of rapamycin
TSE	Transmissible spongiform encephalopathies
TSC	Tuberous sclerosis complex
UPS	Ubiquitin-proteasome system
Ulk1	Unc-51-like kinase 1
VPA	Valproic acid
